# Advanced Nonvolatile Organic Optical Memory Using Self-Assembled Monolayers of Porphyrin–Fullerene Dyads

**DOI:** 10.1021/acsami.1c24979

**Published:** 2022-03-28

**Authors:** Lyubov A. Frolova, Yulia Furmansky, Alexander F. Shestakov, Nikita A. Emelianov, Paul A. Liddell, Devens Gust, Iris Visoly-Fisher, Pavel A. Troshin

**Affiliations:** † Institute for Problems of Chemical Physics of Russian Academy of Sciences,Semenov av. 1, Chernogolovka, Moscow Region 142432, Russia; ‡ Yersin Department of Solar Energy & Environmental Physics, Blaustein Institutes for Desert Research, Ben-Gurion University of the Negev, Sede Boqer Campus, Midreshet Ben Gurion 8499000, Israel; § School of Molecular Sciences, College of Liberal Arts and Sciences, Arizona State University, Tempe, Arizona 85287-1604, United States; ∥ Silesian University of Technology, Akademicka 2A, 44-100 Gliwice, Poland

**Keywords:** porphyrin−fullerene dyad, self-assembled monolayer, organic field-effect transistors, OFETs, optical memory, photoswitching

## Abstract

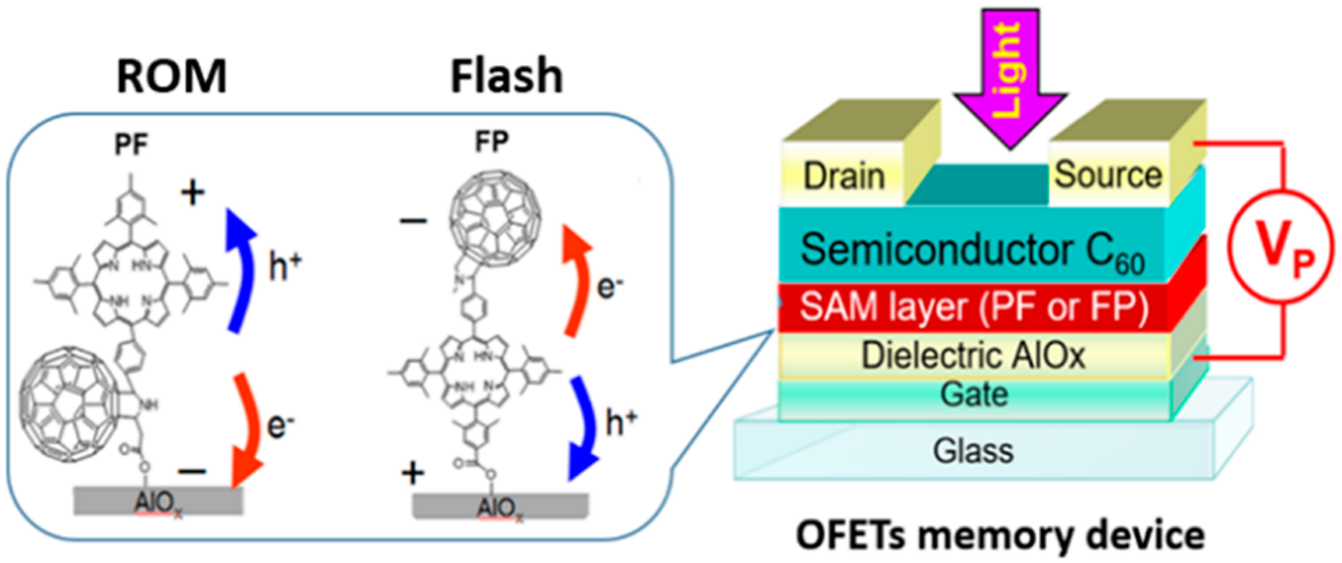

Photo-switchable organic field-effect transistors (OFETs) represent an important platform for designing memory devices for a diverse array of products including security (brand-protection, copy-protection, keyless entry, etc.), credit cards, tickets, and multiple wearable organic electronics applications. Herein, we present a new concept by introducing self-assembled monolayers of donor–acceptor porphyrin–fullerene dyads as light-responsive triggers modulating the electrical characteristics of OFETs and thus pave the way to the development of advanced nonvolatile optical memory. The devices demonstrated wide memory windows, high programming speeds, and long retention times. Furthermore, we show a remarkable effect of the orientation of the fullerene–polymer dyads at the dielectric/semiconductor interface on the device behavior. In particular, the dyads anchored to the dielectric by the porphyrin part induced a reversible photoelectrical switching of OFETs, which is characteristic of flash memory elements. On the contrary, the devices utilizing the dyad anchored by the fullerene moiety demonstrated irreversible switching, thus operating as read-only memory (ROM). A mechanism explaining this behavior is proposed using theoretical DFT calculations. The results suggest the possibility of revisiting hundreds of known donor–acceptor dyads designed previously for artificial photosynthesis or other purposes as versatile optical triggers in advanced OFET-based multibit memory devices for emerging electronic applications.

## Introduction

The photoactive dyads, formed by linking electron-donor organic molecules such as porphyrins with electron-acceptor fullerene derivatives, have been attracting much attention for the last two decades as promising functional materials for organic optoelectronic devices.^1−3^ The donor–acceptor dyads can undergo efficient intramolecular photoinduced charge separation, which has inspired intense research on the application of these materials in artificial photosynthesis^4−6^ and photoelectrochemical cells for solar energy conversion.^1,2,7−19^ Hundreds of exciting molecular assemblies have been designed and investigated, and extraordinarily long lifetimes of charge-separated states have been reported.^20−22^ These fundamentally important findings have not as yet resulted in any commercialized technologies, and the focus of the research has gradually shifted to more conventional photovoltaic concepts such as organic and more recently also perovskite solar cells.^23,24^

Since donor–acceptor dyads represent molecular light-triggered switches, there is a huge and largely unexplored potential for their application in optoelectronics, particularly in the design of memory devices. Among the many possible architectures of organic memories, organic field-effect transistors (OFETs) represent one of the most promising platforms for the design of affordable, flexible, and lightweight data storage devices. Currently, nonvolatile organic memory devices are actively being developed using floating-gate OFET architectures,^25−27^ transistors with charge-trapping layers of polymeric electrets,^28−31^ or ferroelectric components.^32−36^ However, despite the tremendous progress achieved in this field, most of the demonstrated organic memories operate at relatively high voltages (tens of volts), require long programming times (seconds to minutes), and rarely demonstrate retention times exceeding 10^5^ s.

Given the fact that the highest density of the charge carriers in an operating OFET is carried in a few molecular layers of the semiconductor adjacent to the interface with the dielectric,^37^ changing the properties of this interface can induce significant modulation of the device electrical characteristics. Recently, we have explored this approach and introduced photochromic molecules at the semiconductor/dielectric interface. The organic photochromic compounds served as molecular triggers, which could be modulated using different optoelectrical programming regimes to enable advanced organic memory elements operating with high switching coefficients of ∼10^4^–10^5^ at reasonably low operation voltages (3–10 V). Such devices demonstrated good write–read–erase cycling endurance as well as data retention times exceeding 1 month.^38,39^ Unfortunately, the writing speeds were still relatively slow (10–30 ms), which leaves substantial room for further improvements.

Herein, we present a new concept for designing advanced organic memories using monolayers of porphyrin–fullerene dyads placed at the semiconductor/dielectric interface in OFETs. We demonstrate for the first time that donor–acceptor dyads can be used as molecular triggers enabling strong photoswitching of OFETs which leads to the formation of multiple distinct and stable electrical states as required for memory applications. Furthermore, it was found that the molecular structure of the fullerene-porphyrin dyad governs the device programming behavior, thus enabling the design of both flash and ROM types of memory with advanced electrical characteristics.

## Experimental Section

### Characterization of the Porphyrin–Fullerene PF and FP Dyads Molecular Layers

Adsorption of the dyads on an AlO_*x*_ gate electrode surface was confirmed by contact angle and absorption spectroscopy measurements. The water contact angle increased from ca. 1° to ca. 90° ± 2° indicating the formation of dyad layers, with only a minor reduction (<5°) after thorough sonication in mesitylene. Quantitative characterization of the dyad layer density was performed by desorption of the dyads from electrodes of a known area in mesitylene solution containing 3% (*v*/*v*) of trimethylamine and 10% (*v*/*v*) of ethanol. The shape of the UV–vis spectrum of the desorbed dyad matched the spectrum of the pristine material in solution (Figure S1). Using the dyads’ extinction coefficients in mesitylene solutions (487 807 M^–1^cm^–1^ for FP-dyad and 568 948 M^–1^cm^–1^ for PF-dyad at 420 nm, according to our calibration) and the measured absorbance, the molecular density of the dyads adsorbed on AlO_*x*_ was estimated to be about 7.4 × 10^13^ molecules per cm^2^, which is comparable with the literature values reported for carboxylic acid induced monolayers adsorbed on TiO_2_ and AlO_*x*_ surfaces.^40,41^ We therefore conclude that the adsorbed layer is likely to have a density in the order of a monolayer.^41^

To evaluate the uniformity of the absorbed on AlO_*x*_ molecular layers of PF- and FP-dyads, scattering-type scanning infrared near-field optical microscopy (IR s-SNOM, Neaspec, Germany) has been applied. This technique provides the local infrared spectroscopy analysis with the lateral resolution of ca. 25–35 nm. In the context of our study, IR s-SNOM could directly visualize the distribution of the dyad layer on the AlO_*x*_ surface. The IR s-SNOM images obtained by scanning at the IR absorption frequencies of PF and FP dyads (1608 and 1691 cm^–1^, respectively; FTIR spectra are shown in Figure S2) revealed that both of them form uniform coatings over the AlO_*x*_ films without any voids or pinholes as indicated by homogeneous red color distribution on the corresponding images in Figure S3. At the same time, the reference blank aluminum oxide films showed virtually now s-SNOM signal on these frequencies.

### Fabrication and Characterization of the Memory Device

The glass substrates were first sonicated in piranha solution (a mixture of H_2_O_2_ and H_2_SO_4_) for 5 min, then are thoroughly rinsed in deionized water and dried in an oven at 60 °C for 20 min with subsequent RF plasma treatment (150 W) for 5 min. Aluminum gate electrodes with a thickness of 200 nm were deposited by thermal evaporation in a vacuum (2 × 10^–6^ mbar) through a shadow mask. A thin layer of AlO_*x*_ (∼10 nm) was grown via electrochemical anodic oxidation of aluminum gate electrodes in 0.1 M citric acid (Acros Organics) at a constant potential of 12 V as described previously.^39^ Afterward, the monolayers of the porphyrin–fullerene dyads were deposited by immersing the substrates in a ∼0.5 mM PF or FP dyads solution in mesitylene for 30 min. Afterward, the samples were vigorously rinsed with pure mesitylene to remove the excess of PF or FP dyads, which did not bond to the AlO_*x*_. Then the samples were transferred to the vacuum evaporator chamber integrated into the glovebox and fullerene C_60_ was thermally deposited with a rate of 0.3 Å s^–1^ at 320 °C under high vacuum (2 × 10^–6^ mbar) to form an 80 nm thick semiconductor layer. Finally, 100 nm silver source and drain electrodes were thermally evaporated through a shadow mask defining the device geometry with the channel length and width of 60 μm and 2 mm, respectively.

The electrical characterization of OFETs was carried out in an MBraun glovebox in an inert argon atmosphere (H_2_O, O_2_ < 0.1 ppm) using a double-channel Keithley 2612A instrument. A diode laser with a power of 60 mW and a sharp maximum at 405 nm modulated with Advantest R6240A were used for programming the memory elements.

### Density Functional Theory (DFT) Calculations

To calculate the structure and properties of the dyads, quantum chemical calculations were carried out using the PBE density functional method^42^ with SBK pseudopotential^43^ and an extended basis set for valence shells implemented in the PRIRODA software package.^44^ Atomic charges were determined by Hirshfeld charge analysis.^45^ All calculations were performed using the facilities of the Joint Supercomputer Center of the Russian Academy of Sciences. Details are given in Supporting Information.

## Results and Discussion

**Figure 1:**
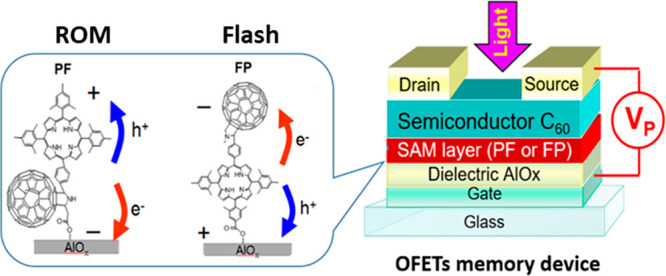
Molecular structures of the investigated porphyrin–fullerene dyads with anchoring carboxylic groups attached to the fullerene (PF) or the porphyrin (FP) units thus enabling their different alignment on the dielectric oxide layer (left). Schematic layout of the device architectures incorporating photosensitive monolayers of PF or FP (right).

We have investigated two structurally similar fullerene-porphyrin dyads FP and PF, which have the surface-anchoring carboxylic groups attached to either the porphyrin or the fullerene moieties, respectively (Figure 1). The synthesis and characterization of the porphyrin–fullerene dyads were reported previously.^46,47^ The molecular formulas of PF and FP enable their different orientations within the self-assembled monolayers as shown in Figure 1. The porphyrin moieties of the FP dyad are linked to the oxide surface, whereas the fullerene part is bonded to AlO_*x*_ in the case of the PF dyad. These different arrangements of the donor–acceptor dyads on the oxide surface result in completely different electrical behavior of the devices as will be discussed below. Both PF and FP dyads were investigated as light-sensitive components integrated into the structure of OFETs at the semiconductor/dielectric interface (Figure 1).

The performance of the fabricated OFETs as memory elements was evaluated by using a hybrid optoelectrical programming regime based on the simultaneous application of an electrical bias between the source and gate electrodes (programming voltage, *V*_P_) and the illumination of the channel of the device with violet light (λ = 405 nm, light intensity 60 mW/cm^2^) as shown schematically in Figure 1. The device behavior was monitored by measuring the transfer characteristics after each programming step.

**Figure 2:**
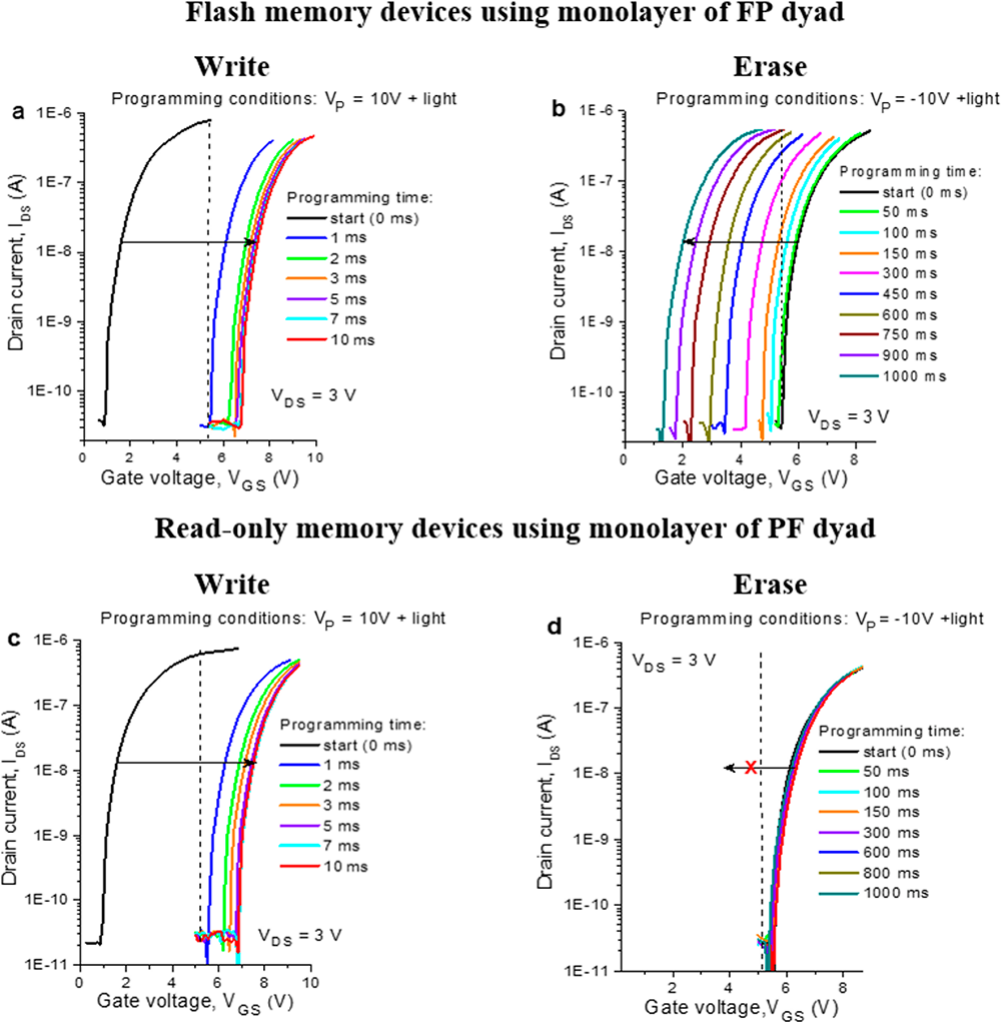
Evolution of the transfer characteristics of the devices comprising FP (a, b) or PF (c, d) dyads under exposure to positive (writing: *V*_P_ = 10 V; a, c) or negative (erasing: *V*_P_ = −10 V; b,d) applied bias and violet light (λ = 405 nm) as a function of the programming time.

To investigate the programming speed of the fabricated memory devices, they were exposed to laser pulses with durations varied between 0.5 and 1000 ms while simultaneously applying *V*_P_ bias. Figure 2 shows the evolution of the transfer characteristics of the OFETs comprising FP or PF dyads upon such programming. When a positive *V*_P_ bias (+10 V) in combination with light is applied, both types of devices demonstrate a rapid increase in the threshold voltage (*V*_TH_) (Figure 2a,c).

The programming within 1 ms is already sufficient to induce a considerable photoswitching effect: *V*_TH_ shifts by ∼4 V and the drain current (I_DS_) monitored at the constant gate voltage *V*_GS_ = 5.3 V is decreased by more than 5 orders of magnitude. Impressive switching coefficients *k*_SW_ = *I*_DS_(state 1)/*I*_DS_(state 2) approaching ∼2.6 × 10^5^ and ∼2.2 × 10^5^ were obtained for the devices comprising FP or PF, respectively. Programming with the negative *V*_P_ bias (−10 V) and light enables a reverse transition of the device transfer characteristics accompanied by a decrease in *V*_TH_ value for the OFETs incorporating the FP dyad (Figure 2b).

However, the speed of the backward transition was found to be considerably lower: Programming for about 1 s was required to return the FP-based devices to the initial state, whereas 150 ms is sufficient for device switching with *k*_SW_ exceeding 10^3^ (Figure S4). Thus, the OFETs loaded with the monolayer of the FP dyad can operate as flash memory devices supporting the recording (writing) of information at positive *V*_P_ and the erasing at negative *V*_P_. Surprisingly, the devices incorporating the PF dyad were completely insensitive to applying negative programming bias and light (Figure 2d). Thus, the programming of PF-based devices with a positive *V*_P_ bias was essentially irreversible. In other words, the information recorded in the memory cells by their exposure to the positive *V*_P_ and light cannot be erased, so using the PF dyad enables the fabrication of so-called read-only memory (ROM) devices. Thus, the differences in the molecular geometry of the FP and PF dyads and the ways of their attachment to the oxide dielectric result in large effects on the electrical behavior of the devices and enable the fabrication of both flash and ROM memory devices.

**Figure 3:**
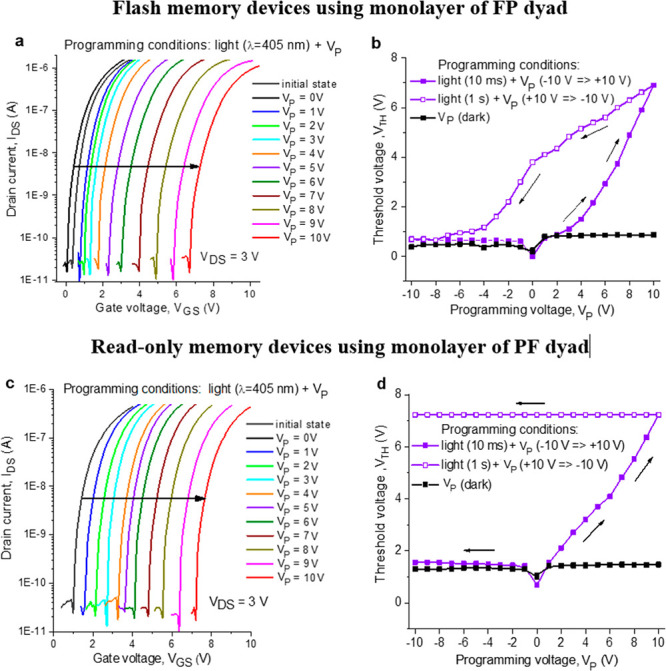
Evolution of the transfer characteristics of the OFETs comprising FP (a) or PF (c) induced by applying gradually increasing *V*_P_ (from 0 to 10 V) and violet light (λ = 405 nm) for 10 ms. Evolution of the OFET threshold voltage as a function of the programming voltage *V*_P_ for OFETs assembled using monolayers of FP (b) and PF (d) dyads. The backward programming was done by applying gradually decreasing *V*_P_ (from +10 to −10 V) under simultaneous exposure to violet light (λ = 405 nm) for 1 s at each step.

Variation of the programming conditions can be used to induce multiple discrete electrical states in the OFETs comprising FP or PF dyads. Figure 3a,c shows the shifts of the transfer curves of the devices upon exposure to different programming voltages ranging from 0 to 10 V combined with the fixed laser pulse duration (10 ms). The device threshold voltage increases from ∼0 to ∼7 V in the case of FP-based OFETs and from ∼1 to ∼7 V for OFETs with PF.

The returning of the devices loaded with the FP dyad (*V*_TH_ shifts from ∼7 to ∼0.5 V) to the initial state can be accomplished by applying negative *V*_P_ biases (from 0 V down to −10 V) and light in a stepwise mode with a 1 s exposure time at each step. It is worth noting that the forward (positive *V*_P_) and backward (negative *V*_P_) transitions of the devices incorporating the FP dyad occur with some hysteresis as shown in Figure 3b. This behavior was found to be highly reproducible for multiple cycles (see below); therefore, it does not complicate the operation of the devices as reliable memory elements. The observed hysteresis appeared simply because the backward device transition requires a higher negative voltage amplitude (and usually longer time) as compared to the forward transition. The devices comprising the PF dyad showed no signs of the backward transition under the applied negative bias voltages and light (Figure 3d), which confirms that the induced electrical states cannot be erased as is typical of ROMs.

It should be emphasized that the simultaneous action of light and electric bias is crucial for switching the OFETs loaded with either PF or FP dyads. Indeed, applying electrical bias *V*_P_ without light causes only negligible *V*_TH_ shifts of the transistors, as illustrated by black lines in Figures 3b,d and sets of current–voltage characteristics presented in Figure S5. Similarly, the exposure of the OFETs to light without electric bias does not lead to any significant changes in their transfer characteristics (Figure S6).

**Figure 4:**
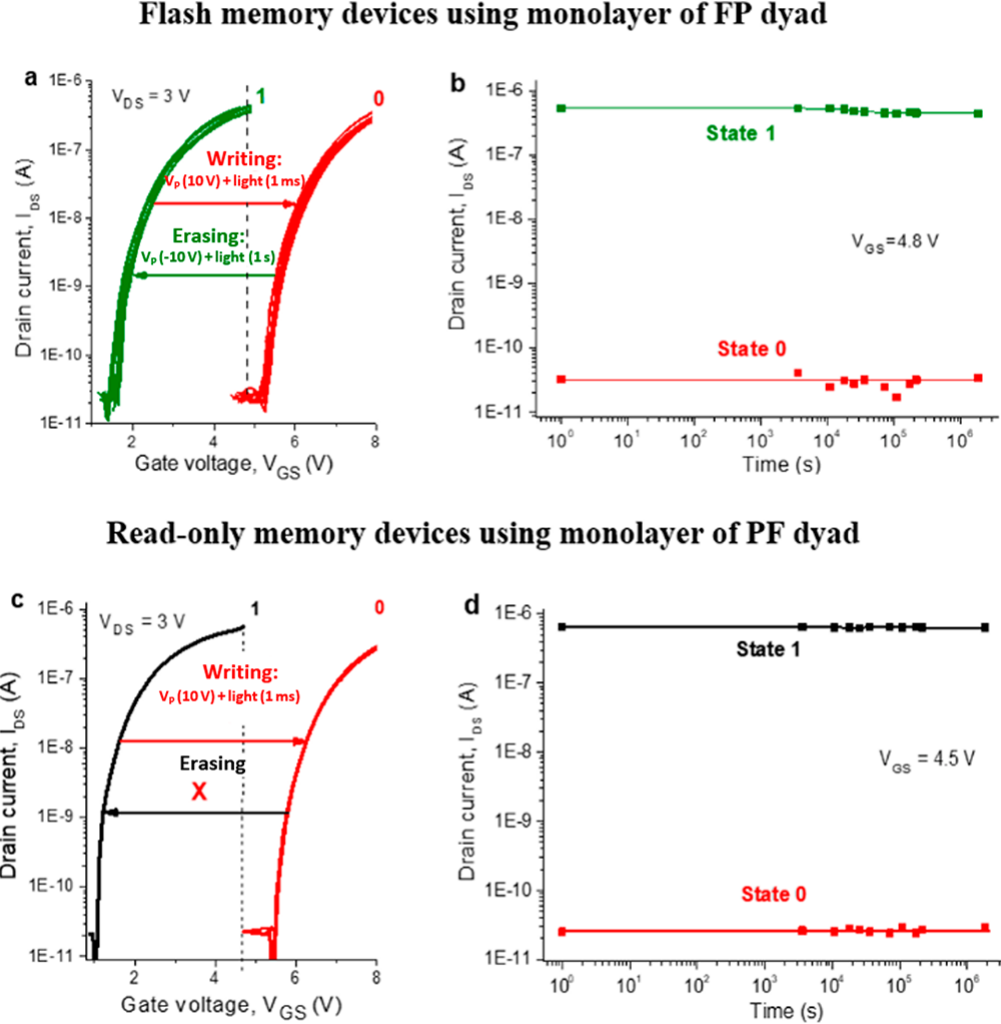
Transfer characteristics illustrating multiple switching of the FP-based OFETs between two distinct electrical states (a) and the irreversible single switching of the devices assembled using the PF dyad (c). OFET drain currents for two distinct electrical states (high-current and low-current) plotted as a function of time illustrate retention characteristics of the memory devices comprising FP (b) or PF (d) dyads.

The retention and endurance characteristics are among the most important parameters of memory devices. The endurance, i.e., the ability to withstand multiple writing and erasing cycles, was investigated only for the devices assembled using the FP dyad as the PF-based OFETs showed irreversible switching behavior (Figure 4c).

In our experiments, we switched the device many times between two arbitrary selected electrical states by simulating the “writing” (*V*_P_ = 10 V + light applied for 1 ms; red lines in Figures 4a and S7) and “erasing” (*V*_P_ = −10 V + light applied for 1 s, green lines) processes. Figure S8 shows 20 manually recorded ‘‘write–read–erase’’ cycles. It is seen from both Figures 4a and S8 that the FP-based devices demonstrate good reproducibility and endurance without any significant degradation in the performance from one cycle to another, which is important for flash memory applications.

To further assess the retention characteristics of the devices incorporating FP or PF dyads, we followed the evolution of the transfer characteristics of the OFETs with time (Figures 4b,d). For both types of devices, we monitored the change of the arbitrarily selected low-current (defined as “0”) and high current (defined as “1”) states in time. Both states were very stable, showing no signs of degradation within at least ∼2 × 10^6^ s, which corresponds to ca. 1 month. Besides, both types of devices showed impressively high and stable “1”/”0” current ratios exceeding 2 × 10^5^ at the read voltage of ∼4.8 V.

**Figure 5:**
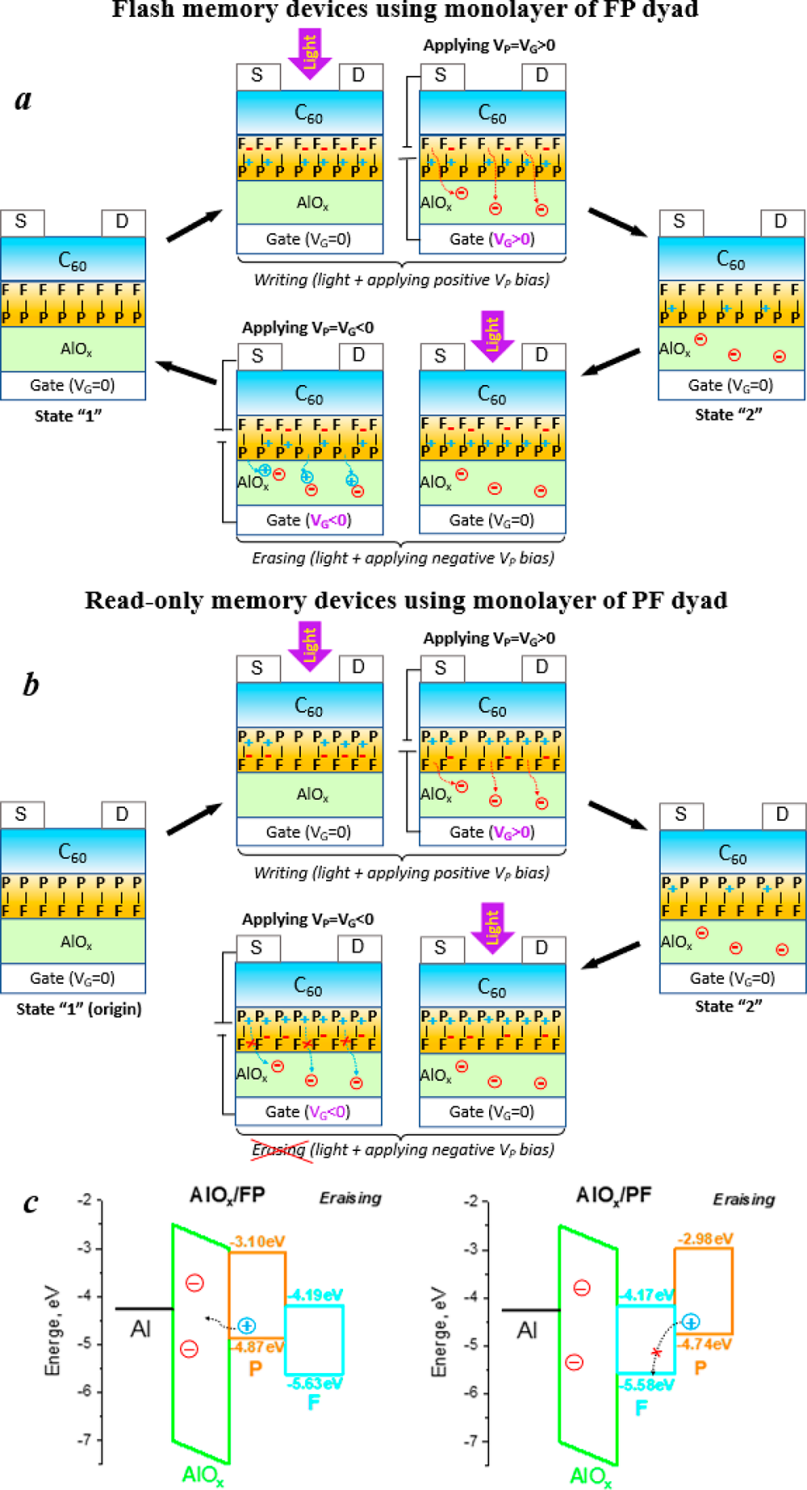
Schematic illustration of the proposed switching mechanisms of the OFETs comprising (a) FP or (b) PF dyads. (c) Energy level diagrams show the facile hole injection from the FP dyad to AlO_*x*_ (left) and the blocked hole injection in the case of the PF dyad (right), as deduced from DFT calculations.

The fundamental differences in the electrical behavior of the OFETs based on the self-assembled monolayers of porphyrin–fullerene dyads PF and FP were very suggestive concerning the operational mechanism of these devices. We assume that at the recording step (positive *V*_P_) the absorption of photons generates charge-separated states in both FP and PF dyads (Figure 5). It should be noted that some light can be partially absorbed in the semiconductor layer, generating C_60_ excitons, which could diffuse to the interface and undergo energy transfer to FP or PF dyad molecules, thus forming the same charge-separated states. The exciton diffusion lengths between 14 and 25 nm were reported for C_60_ and its derivatives.^48,49^

The electric field drives some of the negative charges from the fullerene moieties to the defect sites in the oxide dielectric. Switching off the light results in intramolecular recombination of positive and negative charges except for the dyads which lost their electrons. Therefore, these dyads keep positive charges on the porphyrin units since they cannot recombine with the electrons deeply trapped inside of AlO_*x*_ layer. This electron trapping mechanism is supported by the experimental data since the observed increase in the OFET threshold voltage is fully consistent with the accumulation of positive charge carriers in the transistor channel.

At the erasing step (negative *V*_P_), the light also generates long-living charge-separated states (Figure 5). However, the electric field is driving now-positive charge carriers from the dyads into the dielectric layer toward the negatively biased gate electrode. The injection of holes from the dyads to AlO_*x*_ layer (through some defect sites capable of accommodating positive charge carriers) can readily occur in the case of the FP dyad since the positively charged porphyrin moiety is directly attached to the oxide surface. Therefore, the injected holes recombine with the electrons trapped in the AlO_*x*_ at the recording step, which restores the electrical neutrality of the dielectric layer and converts the device to the initial state. This mechanism explains the flash memory behavior of the OFETs incorporating FP dyad molecules.

In the case of OFETs with PF dyads, on the contrary, injection of holes at the erasing step (negative *V*_P_) is blocked since the porphyrin units bearing the positive charges are separated from the AlO_*x*_ layer by the fullerene moieties. DFT calculations revealed that the highest occupied molecular orbital (HOMO) of the fullerene part of the PF dyad has a much lower energy (−5.58 eV) as compared to the HOMO of the porphyrin unit (−4.74 eV), which makes hole transfer from the porphyrin to the fullerene energetically very unfavorable (Figure 5c). In other words, fullerene cages form a barrier for hole injection from the porphyrins to AlO_*x*_ in the case of OFETs with PF dyad, which does not allow quenching of the negative charges trapped in the dielectric and hence the erasing of the programmed electrical state. In a similar way, other self-assembled monolayers are commonly used in electronic devices to facilitate the injection of one type of charge carriers and block the other.^50,51^ Thus, the proposed mechanism explains why the OFETs incorporating the PF dyad show irreversible switching behavior and operate as read-only memory.

## Conclusion

To summarize, the self-assembled monolayers of donor–acceptor porphyrin–fullerene dyads were applied for the first time as light-sensitive triggers to modulate the optoelectrical switching of organic field-effect transistors and develop nonvolatile optical memory elements. These memory devices demonstrated superior performance characteristics such as low operational voltages, wide memory windows, high current modulation (switching) coefficients approaching 10^5^, fast programming (recording time down to 1 ms), and good endurance and retention characteristics. We found that the position of the anchoring carboxylic group (on the fullerene (PF) or porphyrin (FP) moieties) in the dyads and, consequently, their orientation between the semiconductor and dielectric layers, drastically changes the electrical behavior of the devices. The OFETs comprising interlayers of the FP dyad (anchor group on the porphyrin) showed a reversible photoelectrical switching, which is characteristic for flash memory elements with good write–read–erase cycling stability. On the contrary, the devices based on the PF dyad (anchor group on the fullerene) demonstrated irreversible switching and operated as read-only memory (ROM). The operational mechanism explaining the observed electrical switching behavior of OFETs incorporating FP or PF dyads was proposed and supported by theoretical DFT calculations. Furthermore, both types of memory devices revealed the formation of multiple distinct electrical states and could eventually be used for the development of multibit memory elements capable of high-density information storage required for a variety of practical organic electronics applications. The new concept for the design of advanced organic memory elements demonstrated herein undoubtedly deserves further exploration, in particular by revisiting different donor–acceptor dyads, which were designed and investigated previously as artificial photosynthetic elements.

## Supporting Information Available

Fabrication and characterization methods for porphyrin–fullerene dyads molecular layers and the memory devices, DFT calculations, UV–vis absorption spectra of PF- and FP-dyads, FTIR spectra, IR s-SNOM images, additional memory devices electrical characteristics (PDF)
