# Supplementary material for: Advanced Nonvolatile Organic Optical Memory Using Self-Assembled Monolayers of Porphyrin–Fullerene Dyads
Source: ACS Appl Mater Interfaces. 2022 Mar 28;14(13):15461–7. doi: 10.1021/acsami.1c24979 (PMC8990517; doi:10.1021/acsami.1c24979)
Supplement: Supplementary file 1 — am1c24979_si_001.pdf [file am1c24979_si_001.pdf]

Supporting Information

Advanced non-volatile organic optical memory using self-assembled monolayers of porphyrin-fullerene dyads

*Lyubov A. Frolova[1], Yulia Furmansky[2], Alexander F. Shestakov[1], Nikita A. Emelianov,[1] Paul A. Liddell[3], Devens Gust[3], Iris Visoly-Fisher[2], and Pavel A. Troshin[4,1]**

[1] Institute for Problems of Chemical Physics of Russian Academy of Sciences,

Semenov av. 1, Chernogolovka, Moscow region, 142432, Russia

[2] Yersin Department of Solar Energy & Environmental Physics, Blaustein Institutes for Desert Research, Ben-Gurion University of the Negev, Sede Boqer Campus, Midreshet Ben Gurion 8499000, Israel

[3] School of Molecular Sciences, College of Liberal Arts and Sciences, Arizona State University, Tempe, AZ 85287-1604, USA

[4] Faculty of Chemistry, Silesian University of Technology, Strzody 9, 44-100 Gliwice, Poland

* Corresponding author E-mail: troshin2003@inbox.ru

Density Functional Theory (DFT) calculations

Fullerene-porphyrin dyads FP and PF differ in the position of the anchoring carboxylic group, which is attached to the donor porphyrin moiety in FP and the acceptor fullerene unit in PF. To take into account these differences, we will denote below the FP and PF dyads as A1D1 and D2A2, respectively.

To calculate the structure and properties of the dyads, quantum chemical calculations were carried out using the PBE density functional method[1] with SBK

pseudopotential[2] and an extended basis set for valence shells implemented in the PRIRODA software package.[3] Atomic charges were determined by Hirshfeld charge analysis.[4] All calculations were performed using the facilities of the Joint Supercomputer Center of the Russian Academy of Sciences. The computed structures of the dyads A1D1 and D2A2 are shown in Fig. T1.

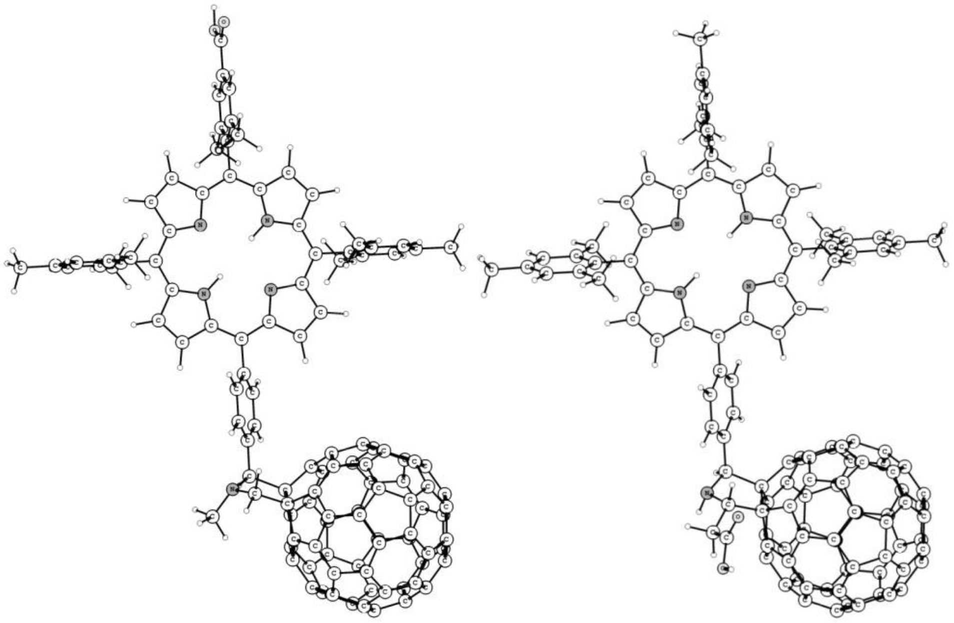

(a) (b)

Figure T1. Computed structures of the dyads FP (A1D1) (a) and PF (D2A2) (b).

The introduction of the carboxylic group slightly affects the frontier energy levels of individual components, therefore the HOMO energy level of D1H is 0.07 eV lower than that of D2H, whereas the LUMO energy level of A2H is 0.02 eV lower than that of A1H. Similarly, the electron affinity of A2H is 0.03 eV higher than that of A1H, and the ionization potential of D1H is 0.12 eV higher than that of D2P (see Table T1).

Table T1. Energies of the boundary orbitals (HOMO, LUMO), ionization potentials PI, electron affinity EA, the energy of triplet-singlet splitting E_{TS}, and dipole moments in the ground (D) and the triplet (D_T) states

System	HOMO, eV	LUMO, eV	PI, eV	EA, eV	$E_{TS,}$ eV	D, Debye	D_T, , Debye
A1H	-5.63	-4.19		2.83	1.16	4.01	4.52
D1H	-4.87	-3.10	6.23			3.26	
A1D1	-4.92	-4.15	6.14	2.87	1.21	4.18	10.01

A2H	-5.58	-4.17		2.86	1.17	3.66	4.10
D2H	-4.74	-2.98	6.11			0.07	
A2D2	-4.79	-4.18	6.05	2.85	1.19	3.86	13.23

There is an insignificant transfer of electron density between the donor and acceptor components of the dyads in the ground state. Therefore, the HOMO levels of the dyads are very close to the HOMO levels of the donor components, while the LUMO levels of the dyads match the LUMO levels of the acceptor components. Nevertheless, the observed differences correlate with the magnitude of the charge localized on the fullerene component: 0.092 in A1D1 and 0.076 in D2A2. When removing the porphyrin component, the charge is -0.044 and -0.035 for A1H and A2H, respectively.

An increase in the negative charge density on the donor component manifests itself in a decrease in the ionization potential by 0.09 and 0.06 eV for the dyads A1D1 and D2A2 as compared to D1H and D2H, respectively. This effect is greater when there is a carboxyl group attached to D1. A similar increase in the electron affinity of the dyads compared to the A1H and A2H were also observed, but these effects were less pronounced.

Since the electronic excitation is localized on the fullerene component, the energies of the singlet-triplet transition E_{TS} in the dyads are noticeably larger than the energy gap between the boundary orbitals and are close to the values of E_{TS} in A1H and A2H. The appearance of a hole in the filled orbitals of the fullerene component promotes the transfer of the electron density from the donor component to the acceptor component in the triplet state. This transfer is 0.178 e in D2A2 and 0.148 e in A1D1, which leads to a significant increase in the dipole moment of the dyads in the triplet state correlating with the magnitude of the transferred charge.

The presence of electronic interactions between the components of the dyads is manifested when they accommodate either an electron or a hole. In negatively charged species, most of the additional electron density is localized on the acceptor component, 82.8 and 79.1% for D2A2 and A1D1, respectively. In positive ions, most of the electron density leaves the donor component, 76.4 and 73.3% for D2A2 and A1D1, respectively. This also correlates with the enhancement of the acceptor strength of A2 and D1 due to the presence of the appended electron-withdrawing carboxylic group.

The performed computational analysis illustrates that there are subtle differences in the electronic characteristics of the two dyads due to the electron-withdrawing effect of the

carboxylic group, which enhances the acceptor strength of the fullerene component and, conversely, decreases the donor strength of the porphyrin component.

Additional results

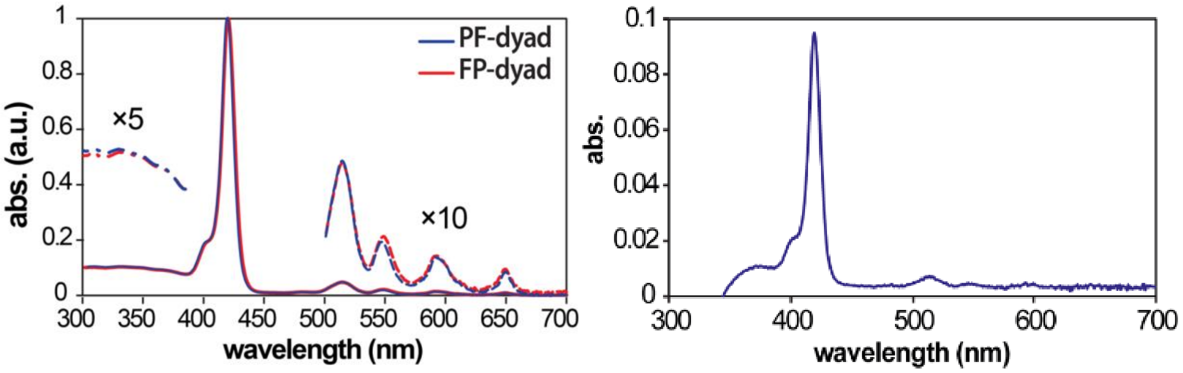

Figure S1. UV-vis absorption spectra of PF- and FP-dyads: (left) dissolved in mesitylene solutions (normalized). Dashed and dashed-dotted lines indicate magnification of the Q-band and fullerene absorption regions, respectively; (right) dissolved in mesitylene from 2.34 cm^2 AlO_x electrode surface.

a

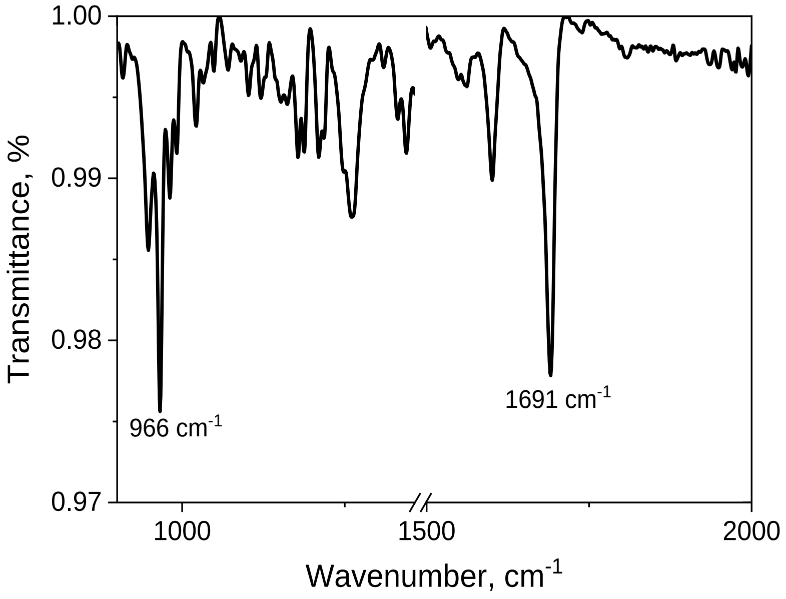

b

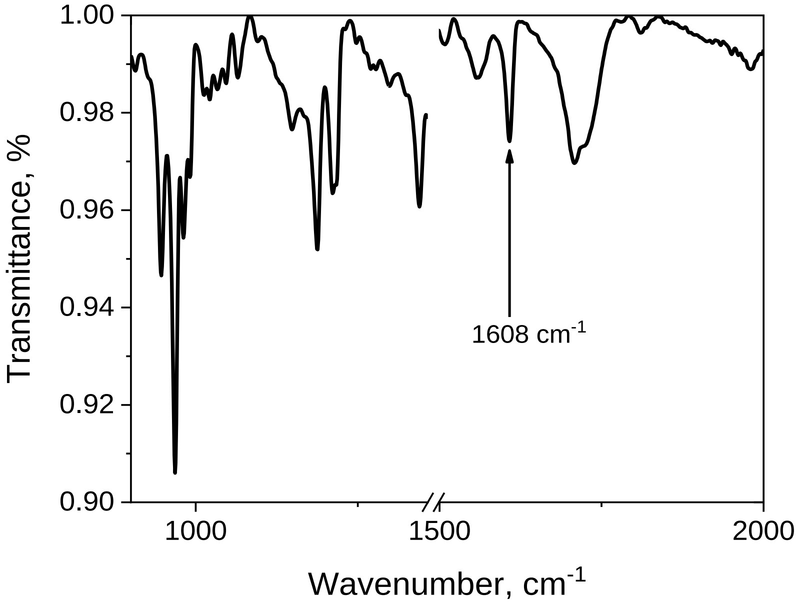

Figure S2. Infrared spectra of the individual powders of FP (a) and PF (b) dyads.

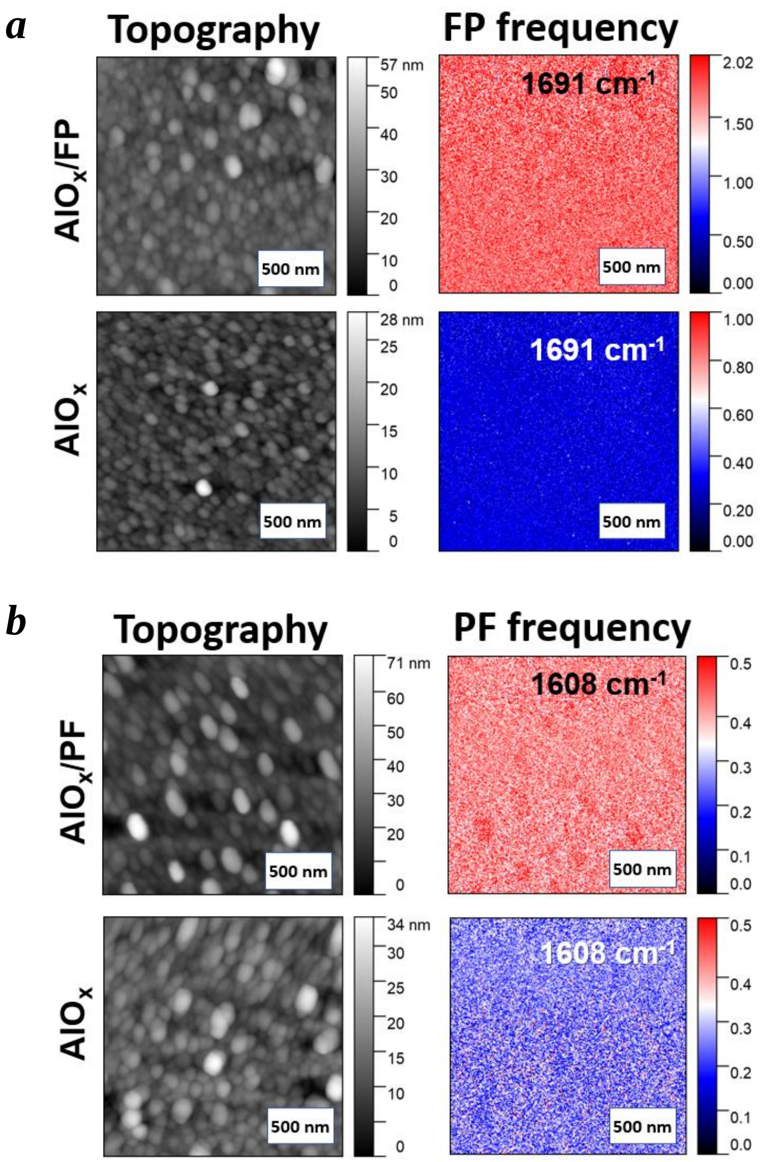

Figure S3. AFM topography (left column) and IR s-SNOM amplitude mapping images for the glass/Al/AlO_x/ FP dyad (a) and glass/Al/AlOx/ PF dyad samples (b) compared to the reference blank glass/Al/AlO_x samples recorded at the corresponding IR absorption frequencies of the dyads (right column).

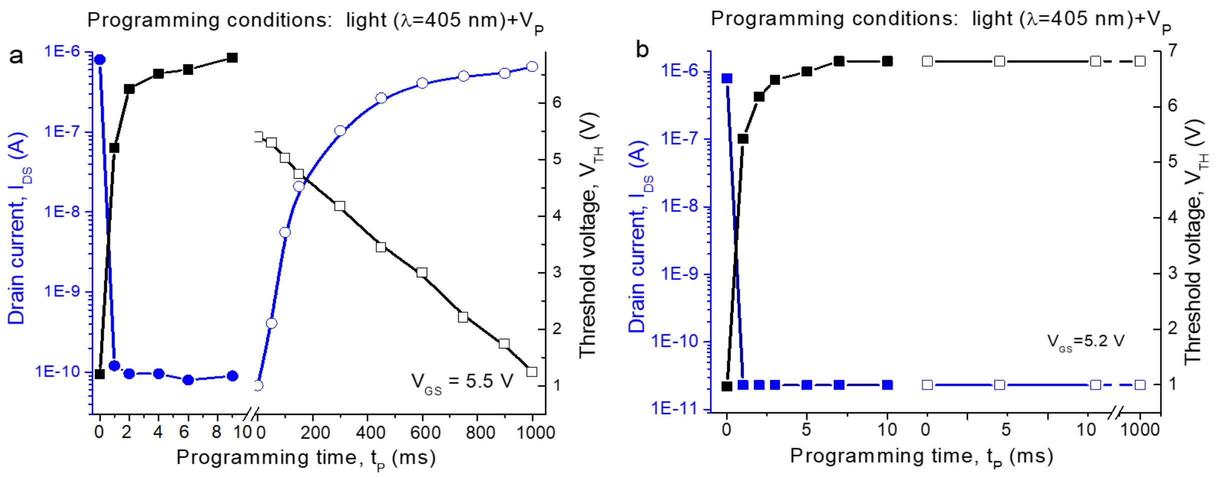

Figure S4. Evolution of the OFET drain currents (circles) and threshold voltages (squares) while programming the devices comprising FP (a) or PF (b) first with the positive (V_p=10V, closed symbols) and then with the negative (V_p=-10V, open symbols) electric bias coupled with simultaneous exposure to light (λ=405 nm).

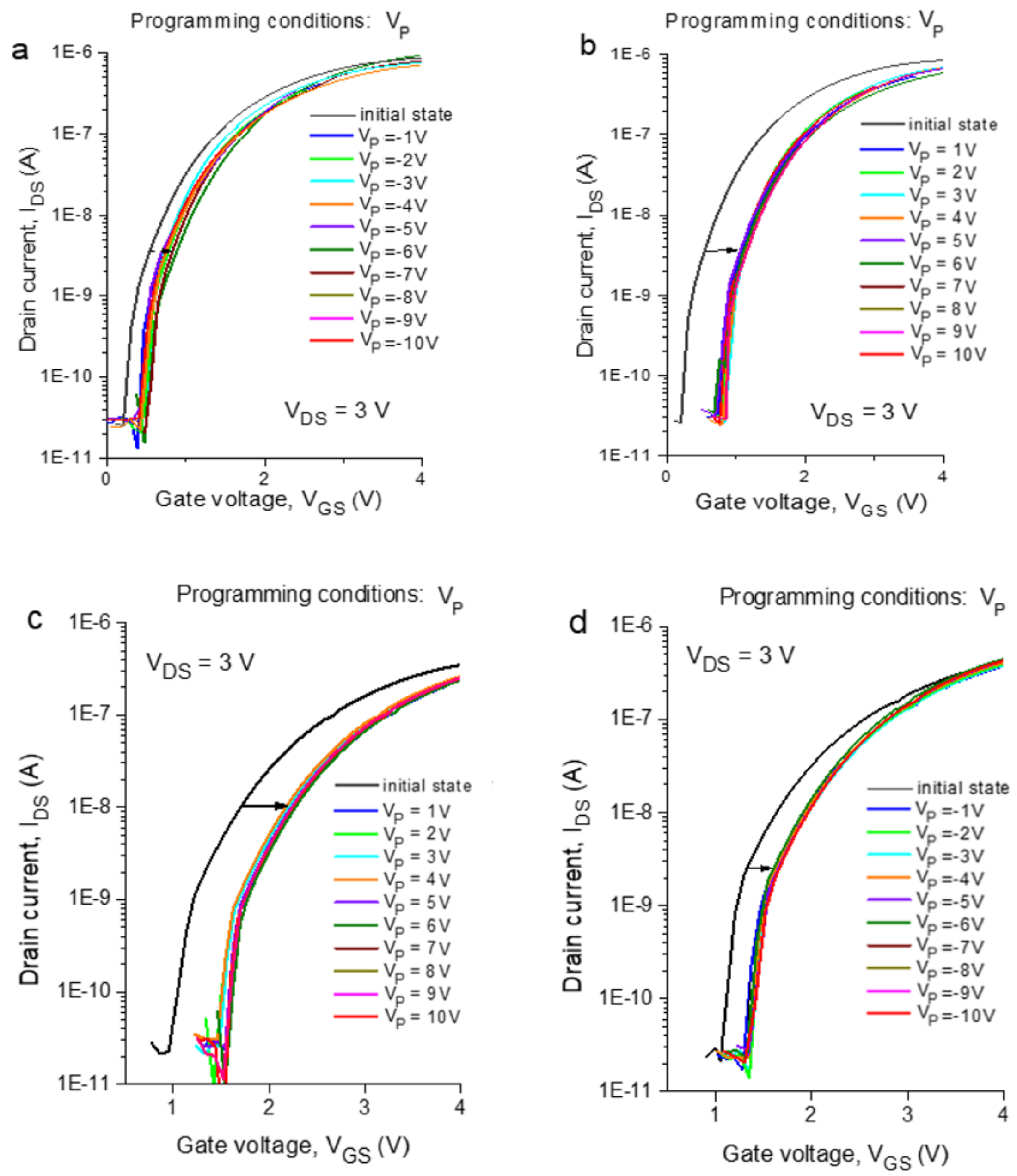

Figure S5. The evolution of the transfer characteristics of the OFETs comprising FP (a, b) or PF dyads (c, d) under exposure to the gradually decreasing (from 0 V to -10 V) or increasing (from 0 V to 10 V) V_P bias potentials in the dark.

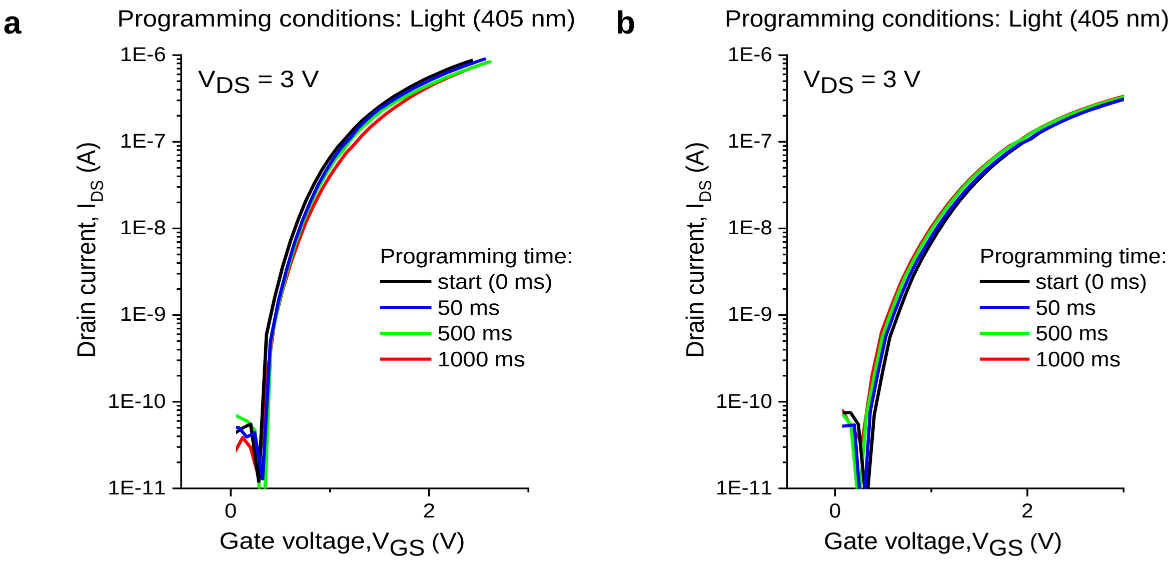

Figure S6. The evolution of the transfer characteristics of the OFETs comprising FP (a) or PF dyads (b) under exposure to light (λ=405 nm) without applying electric bias V_P

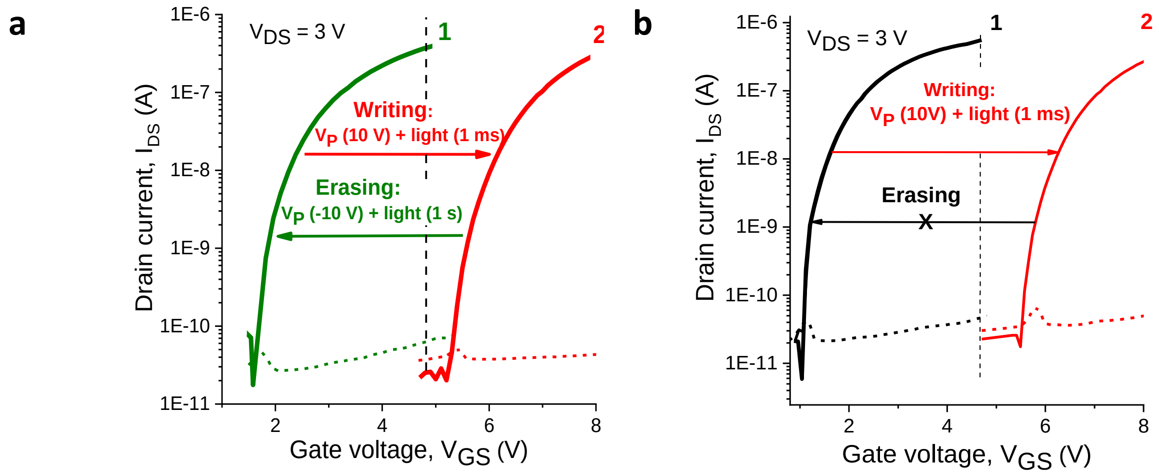

Figure S7. Transfer characteristics and the corresponding leakage currents illustrating the switching of the FP-based OFETs between two distinct electrical states (a) and the irreversible single switching of the devices assembled using the PF dyad (b).

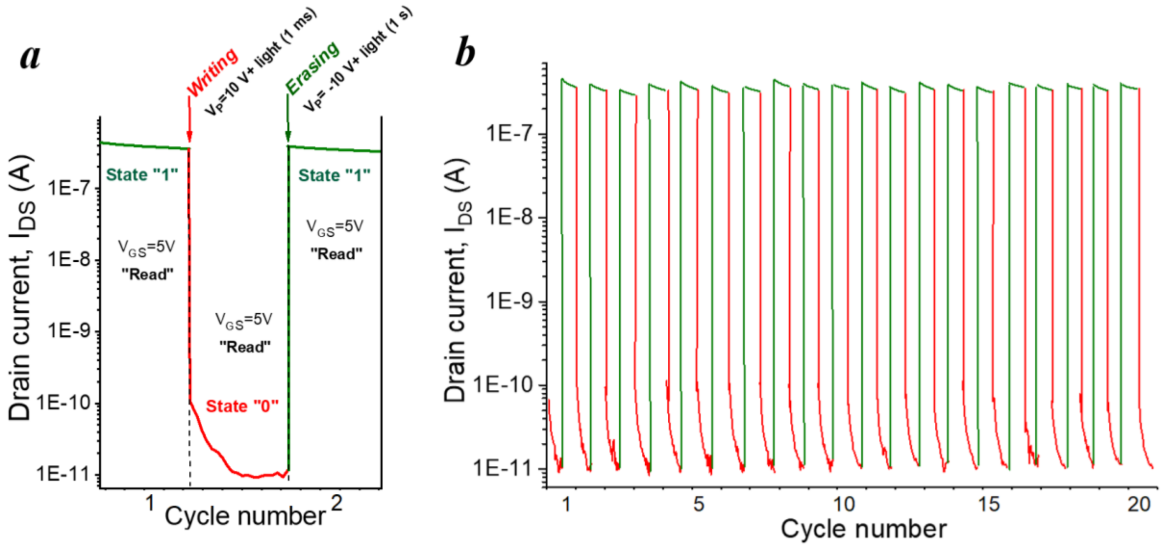

Figure S8. Illustration of the write/erase process (a) and write–read–erase cycling behavior of the OFET-based memory devices comprising FP dyad (b).

References:

(1) Perdew, J. P.; Burke, K.; Ernzerhof, M. Generalized Gradient Approximation Made Simple. *Phys. Rev. Lett.* **1996**, *77* (18), 3865–3868. https://doi.org/10.1103/PhysRevLett.77.3865.

(2) Stevens, W. J.; Basch, H.; Krauss, M. Compact Effective Potentials and Efficient Shared-exponent Basis Sets for the First- and Second-row Atoms. *J. Chem. Phys.* **1984**, *81* (12), 6026–6033. https://doi.org/10.1063/1.447604.

(3) Laikov, D. N. Fast Evaluation of Density Functional Exchange-Correlation Terms Using the Expansion of the Electron Density in Auxiliary Basis Sets. *Chem. Phys. Lett.* **1997**, *281* (1-3), 151–156. https://doi.org/10.1016/S0009-2614(97)01206-2.

(4) Hirshfeld, F. L. Bonded-Atom Fragments for Describing Molecular Charge Densities. *Theor. Chim. Acta* **1977**, *44* (2), 129–138. https://doi.org/10.1007/BF00549096.
